# The melatonin receptor 1B gene links circadian rhythms and type 2 diabetes mellitus: an evolutionary story

**DOI:** 10.1080/07853890.2023.2191218

**Published:** 2023-03-28

**Authors:** Hui Zhu, Zhi-jia Zhao, Hong-yi Liu, Jie Cai, Qin-kang Lu, Lin-dan Ji, Jin Xu

**Affiliations:** a Department of Internal Medicine, Health Science Center, Ningbo University, Ningbo, Zhejiang, P. R. China; b School of Public Health, Health Science Center, Ningbo University, Ningbo, Zhejiang, P. R. China; c Center for Reproductive Medicine, Ningbo Women and Children’s Hospital, Ningbo, Zhejiang, P. R. China; d Department of Ophthalmology, Affiliated People’s Hospital of Ningbo University, Ningbo, Zhejiang, P. R. China; e Department of Biochemistry and Molecular Biology, School of Basic Medical Sciences, Health Science Center, Ningbo, Zhejiang, P. R. China; f Zhejiang Key Laboratory of Pathophysiology, Health Science Center, Ningbo University, Ningbo, Zhejiang, P. R. China

**Keywords:** Melatonin, *MTNR1B*, Type 2 diabetes mellitus, thrifty gene

## Abstract

Disturbed circadian rhythms have been a risk factor for type 2 diabetes mellitus (T2DM). Melatonin is the major chronobiotic hormone regulating both circadian rhythm and glucose homeostasis. The rs10830963 (G allele) of the *melatonin receptor 1B* (*MTNR1B*) gene has the strongest genetic associations with T2DM according to several genome-wide association studies. The *MTNR1B* rs10830963 G allele is also associated with disturbed circadian phenotypes and altered melatonin secretion, both factors that can elevate the risk of diabetes. Furthermore, evolutionary studies implied the presence of selection pressure and ethnic diversity in *MTNR1B*, which was consistent with the “thrifty gene” hypothesis in T2DM. The rs10830963 G risk allele is associated with delayed melatonin secretion onset in dim-light and prolonged duration of peak melatonin. This delayed melatonin secretion may help human ancestors adapt to famine or food shortages during long nights and early mornings and avoid nocturnal hypoglycemia but confers susceptibility to T2DM due to adequate energy intake in modern society. We provide new insight into the role of *MTNR1B* variants in T2DM *via* disturbed circadian rhythms from the perspective of the “thrifty gene” hypothesis; these data indicate a novel target for the prevention and treatment of susceptible populations with the thrifty genotype.

## 1. Introduction

The International Diabetes Federation (IDF) estimated that diabetes affects 537 million adults (20-70 years old) across the world [1]. Type 2 diabetes mellitus (T2DM) is a complex multifactorial metabolic disorder that develops interaction between lifestyle risk factors and genetic susceptibility [2]. Accumulating evidence has demonstrated that the endogenous circadian rhythm, which is responsible for harmonizing the internal clock with the environment and regulating daily behavior (i.e. sleep-wake cycles, feeding and activity), is an additional risk factor for T2DM, as it impacts the diurnal rhythm of glucose metabolism [3–5]. Large cross-sectional and prospective cohort studies have reported that chronic circadian misalignment, such as rotating night-shift work [6–8], social jetlag [9,10], sleep disturbances [6,11] and an evening chronotype [12], confer a higher risk of impaired glucose tolerance, insulin resistance, insufficient β-cell mass and T2DM [13–15].

Melatonin is a critical circadian hormone and has been widely investigated for its complex contribution to sleep quality, circadian regulation and glucose homeostasis [16,17]. Circadian melatonin is mainly secreted from the pineal gland under the control of the hypothalamic suprachiasmatic nucleus (SCN) and environmental light exposure [18,19]. Despite substantial research, whether and how melatonin contributes to glucose metabolism through circadian regulation remains poorly understood. Several studies have mentioned that melatonin protects against glucose tolerance and diabetes mellitus. Large population-based studies within the Nurses’ Health cohort have suggested that lower melatonin release can increase insulin resistance and the incidence of type 2 diabetes [19,20]. Furthermore, a randomized, double-blind clinical trial among type 2 diabetes patients with insomnia also indicated that long-term administration of prolonged-release melatonin could improve glycemic control and inhibit hypertriglyceridemia as well as hyperinsulinemia [21]. Conversely, two other placebo-controlled studies indicated a deleterious effect of acute melatonin administration in the morning or evening on glucose tolerance and insulin sensitivity both in young and older women [22,23].

The contradictory or even completely opposite role of melatonin in glucose metabolism is intriguing. Some researchers have proposed that melatonin receptors may partly explain this contradiction. Melatonin generally exerts its effects through specific, high-affinity G protein-coupled receptors with seven transmembrane domains; these receptors are widely expressed in central and peripheral tissues [24,25], including human islet cells [26,27]. The *MTNR1B* gene, which encodes melatonin receptor 1B, is robustly associated with various glycemic traits, such as increased fasting glucose, aggravated insulin resistance and attenuated β-cell function according to a genome-wide association (GWA) and replicated cohort studies, with T2DM risk most strongly linked to the rs10830963 G allele [28–30]. Notably, this *MTNR1B* gene variant was associated with endogenous melatonin signaling, sleep status and circadian rhythms [31]. Lane et al. previously confirmed that the *MTNR1B* rs10830963 G allele was linked with the prolongation of elevated melatonin levels and delayed offset of melatonin secretion in daytime [32]. Therefore, rs10830963 G allele carriers might have an increased risk of impaired glucose tolerance or T2DM due to elevated melatonin secretion and food intake in the morning. In addition, these risks of rs10830963 allele carriers were more pronounced in individuals with early sleep time [32]. Moreover, T2DM patients carrying rare variants in the *MTNR1B* coding region were more prone to behavioral circadian misalignment and irregular sleep [33]. The most recent randomized crossover trial on this topic revealed that altered behavioral rhythms, such as late dinner time, were obviously associated with 3.5-fold higher melatonin levels; moreover, the effect of late dinners on insulin secretion was stronger in *MTNR1B* rs10830963 G risk allele carriers, which indicates further effects of *MTNR1B* variants on circadian rhythms in terms of β-cell function [34]. These findings all indicate that variants of the *MTNR1B* gene mediate human circadian phenotypes. Additionally, the rs10830963 G allele was linked with the overexpression of *MTNR1B* in human islet cells [35]. The highly expressed G-protein-coupled melatonin receptor was reported to enhance the inhibition of melatonin secretion to enable cyclic adenosine monophosphate (cAMP) synthesis; cAMP is a stimulator of insulin release [18].

Nonetheless, whether and how the circadian rhythm and genetic variants of *MTNR1B* exert separate or joint effects on T2DM *via* the melatonin system are relatively unknown, and the possible mechanisms remain unclear. Circadian misalignment and *MTNR1B* variants appeared to facilitate the development of T2DM in a subset of the population [36]. Supporting this finding, several evolutionary analyses indicated a prominent population difference implying selection pressures on *MTNR1B* in terms of T2DM [37,38]. The present review mainly focuses on the link between the *MTNR1B* gene and T2DM. We first briefly outline the interplay between the circadian and melatonin systems and then elucidate the role of melatonin and its receptor signaling in T2DM *via* the regulation of the circadian rhythm. We also provide an overview of the genetic contribution of *MTNR1B* to T2DM, thus discussing evolutionary selection on *MTNR1B* in terms of glucose homeostasis as well as T2DM, and hypothesize that the *MTNR1B* gene is a “thrifty gene” for humans.

## 2. Melatonin and circadian system

### 2.1. The interplay between melatonin synthesis and circadian rhythms

Various organisms, including humans, have evolved to adapt to the earth’s daily rotation and light/dark cycle. Human ancestors worked (were awake) during the daytime and rested (sleep) at night. Consequently, endogenous biological processes, including physiology, metabolism, and behaviors, accommodated this sleep-wake pattern, which is termed the circadian rhythm or circadian clock [39,40]. In essence, the circadian rhythm is an evolutionarily conserved endogenous autonomous timing system. The human circadian system consists of the central and peripheral clocks. The central clock is located in the SCN of the hypothalamus; it can reset intrinsic circadian rhythms every 24 h and synchronize the peripheral clocks in adipose tissue and the pancreas, liver and gut [41,42]. The accurate molecular mechanism underlying the circadian timing system is executed by transcriptional-translational feedback loops consisting of core clock genes, and the entrained timing signal is ultimately forwarded through neural and hormonal signals [13].

**Figure 1.**
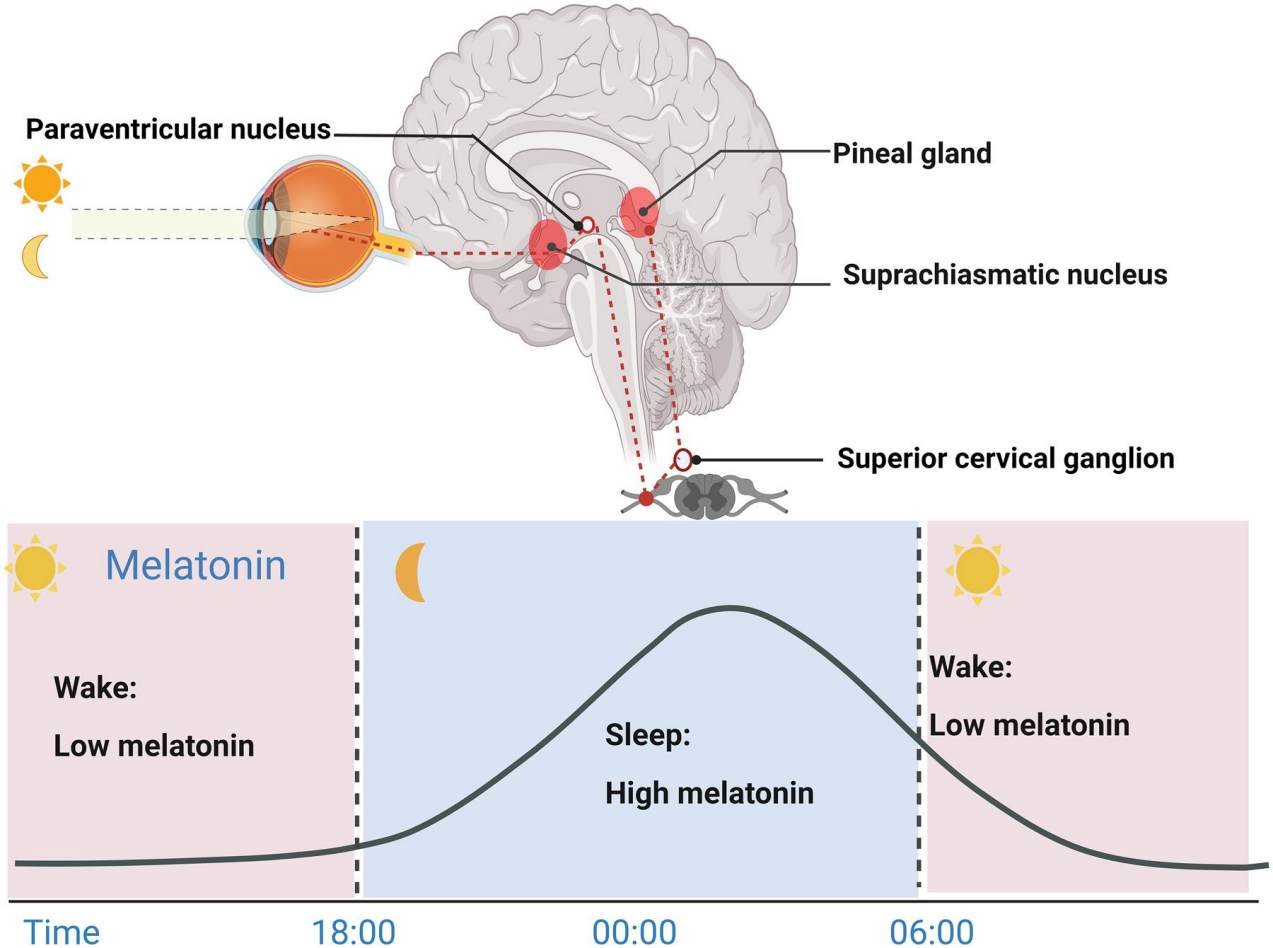
Melatonin levels fluctuate across the 24-h light-dark cycle [41,46,47]. Melatonin synthesis and release occur in dim light and are inhibited by daytime light. In the eyes, environmental light reaches intrinsic photosensitive retinal ganglion cells (ipRGCs) and is then transmitted to the SCN via the retinal hypothalamic tract. SCN signals are conveyed to the medial forebrain bundle by descending hypothalamic projections and then project to the spinal cord and superior cervical ganglia. Afterward, the sympathetic nerve from the superior cervical ganglion stimulates the pineal gland to secrete melatonin, thus entraining circadian rhythms to environmental light. Figure 1 was created with BioRender (https://biorender.com).

Synthesis of the pineal hormone melatonin is regulated by the SCN master clock and synchronized to the environmental light-dark cycle. Melatonin secretion generally occurs in darkness (at night) and peaks at 00:00 and 4:00 am. Importantly, nighttime melatonin production is blocked by light, especially blue light at wavelengths of 460–480 nm and intensities < 200 lux [43–45]. The biosynthetic precursor of melatonin is tryptophan, which is hydroxylated to 5-hydroxytryptophan and then decarboxylated to generate serotonin. Subsequently, serotonin is acetylated to N-acetylserotonin by arylalkylamine N-acetyltransferase (AANAT) and then converted to melatonin by acetylserotonin O-methyltransferase [16]. When the environmental photoperiodic information reaches intrinsic photosensitive retinal ganglion cells (ipRGCs), it is conveyed to the SCN by the retinal hypothalamic tract. Afterward, the signal is projected to the pineal gland through a neuronal signaling cascade that promotes or inhibits melatonin secretion in pinealocytes (Figure 1) [41,46,47].

The SCN-controlled pineal gland is the main source of melatonin production in humans. In turn, pineal melatonin is the key temporal molecule; through feedback, it orchestrates the internal clock to adapt to rhythmic changes in the external light-dark cycle. Once secreted, the neuromodulator melatonin is conveyed to the SCN through the cerebrospinal fluid of the third ventricle or blood circulation system and then binds to specific high-affinity receptors expressed in the SCN [48,49]. It is well established that the binding sites of melatonin in the SCN are essential for transmitting the oscillating signals of the environmental light/dark cycle to the biological clock, subsequently modulating various physiological processes and behaviors [16,49,50]. Hence, melatonin receptors are efficient therapeutic targets for treating circadian abnormalities or sleep disorders [25]. However, a few studies have speculated that melatonin may directly (i.e. not through its receptors) mediate the action of the SCN [50–52]. Moreover, the fluctuating levels of melatonin in the bloodstream affect the circadian rhythm of peripheral tissues. Melatonin shows a rapid pharmacokinetic profile with a half-life of 20–30 min [53]. Therefore, the chronobiotic properties of melatonin in the SCN and peripheral tissues contribute to whole-body synchronization of circadian rhythms.

In summary, melatonin is primarily and indirectly generated by the SCN, consistent with the circadian rhythm, but it is also the key temporal feedback received by the SCN; the feedback loop between the SCN and the neuromodulator melatonin efficiently synchronizes the endogenous circadian rhythm to the photoperiod in humans.

### 2.2. The physiological function of melatonin

Melatonin is a pleiotropic molecule and has multiorgan effects in humans [16,47,54–73] (Table 1). The physiological functions and ensuing effects of melatonin vary in different systems. We primarily focused on its role in metabolic functions in terms of immediate effects, consecutive effects and chronobiotic effects in a previous study [45]. Pineal melatonin was characterized by dim-light melatonin onset (DLMO) and a nocturnal daily peak; melatonin was released in the CSF and blood *via* this classic hormonal pathway and immediately mediated biological function by melatonin signaling [45]. After the cessation of melatonin signaling in the light phase, the intracellular adenylyl cyclase/cAMP/PKA/CREB transduction pathway became hypersensitive, which was inhibited by melatonin signaling in the dark phase. In particular, melatonin facilitates insulin secretion of islet β-cells by directly sensitizing glucagon-like peptide-1 (GLP-1)/AMP/PKA/CREB signaling, attenuating apoptosis of β-cells, diminishing oxidative stress in cells exposed to hyperglycemia and maintaining the glucose-stimulated insulin response [74–77]. Additionally, the chronobiotic property of melatonin allows targeted regulation of peripheral clock gene expression in pancreatic β-cells, adipose tissue, skeletal muscle, liver tissue and other tissues, which are directly involved in multiple metabolic functions [63,78–80]. The melatonin-mediated circadian timing process, including the central and peripheral oscillators, is one of the key regulatory patterns of metabolism and includes several different physiological effects of melatonin.

**Table 1.** The physiological functions of melatonin.

Traits	Physiological functions of melatonin	References
Circadian rhythms and sleep	Melatonin induces phase shift and coordinates circadian rhythms. It also realigns the circadian sleep-wake propensity rhythms by resetting of the circadian system and thermoregulatory mechanisms.	[72–73]
Energy metabolism and obesity	Melatonin establishes an adequate energy balance by regulating the energy expenditure through the activation of brown adipose tissue and participating in the browning process of white adipose tissue. Melatonin synchronizes the circadian rhythms to the activity-feeding-rest-fasting cycle and regulates the glucose uptake in adipocytes.	[54–55]
Fasting plasma glucose (FPG) Type 2 diabetes mellitus (T2DM)	Melatonin maintains the glucose homeostasis by potentiating central and peripheral insulin action due to regulation of GLUT4 expression or triggering the insulin signaling pathway. Melatonin treatment promotes glycaemic control and improves insulin sensitivity in white adipose tissue.	[16,56]
Antioxidant function	Melatonin reduces oxidative stress *via* detoxification of reactive oxygen and reactive nitrogen species, and induces antioxidant enzymes.	[57]
Body temperature	Endogenous melatonin secreted at night generally lower core body temperature (CBT), and this may be associated with the regulatory effects of melatonin on serotonergic or noradrenergic secretion, or vasodilatory changes.	[58–59]
Immune system function	Melatonin displays both pro - and anti -inflammatory properties.	[60–62]
Liver diseases	Melatonin decreases the de novo lipogenesis and liver damage and oxidative and endoplasmic reticulum (ER) stress in liver. Melatonin is associated improved steatosis and insulin resistance, increase of brown adipose tissue mass and activity and restored mitochondria functions.	[63]
Bone loss	Melatonin stimulates osteoblastogenesis, inhibits osteoclastogenesis and improves bone density.	[64]
Cancers	Melatonin decreases tumor size *via* inhibitory effect of angiogenesis in tumor tissues	[69–70]
Cardiocerebral vascular system	Melatonin promotes cardiocerebral vascular system by ameliorating the fatty infiltration into the arterial intima inhibiting the atherosclerosis. It also could decrease elevated blood pressure and promote the stability of atherosclerotic plaques.	[65–68]
Retinal function	Melatonin modulates the activity of photoreceptors in the outer retina.	[47]
Reproductive health	Follicular melatonin levels are positively associated with better ovarian reserve, oocyte quality and antral follicle count, and it also inhibit intra-follicular oxidative stress.	[71]

## 3. Melatonin system influences T2DM *via* circadian rhythms

### 3.1. Circadian rhythms in glucose homeostasis

**Figure 2.**
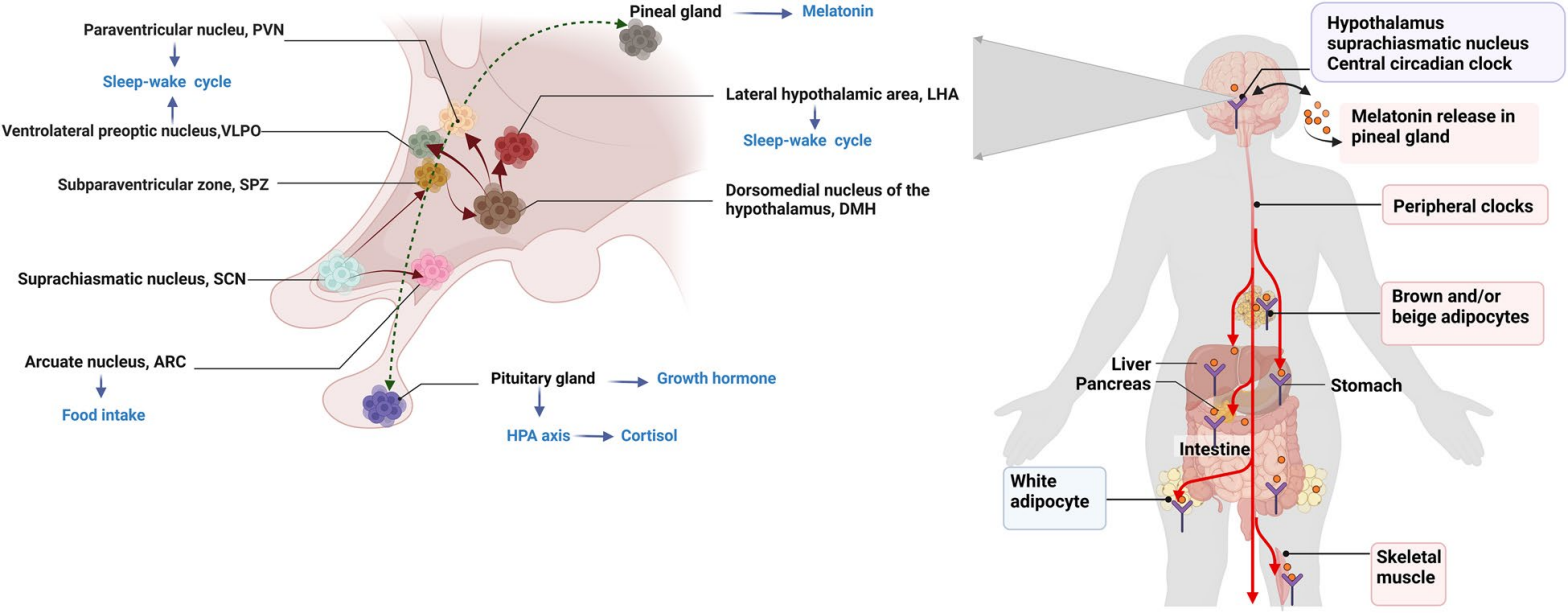
Circadian rhythms maintains glucose homeostasis. Circadian signals are conveyed from the SCN to the adjacent subparaventricular zone (SPZ), and the input is then integrated and amplified in the dorsomedial nucleus of the hypothalamus (DMH). Neurons in the DMH relay information to the ventrolateral preoptic nucleus (VLPO), the lateral hypothalamic area (LHA), orexin receptors and the paraventricular nucleus (PVN), which drive the circadian cycles of sleep, activity, feeding and corticosteroid secretion, respectively. The SCN could directly exerts excitatory-inhibitory effects on the neuronal response of the arcuate nucleus (ARC) to hypoglycemia and modulate food intake. The central circadian clock synchronizes peripheral clocks, and jointly regulate glucose metabolism. Figure 2 was created with BioRender (https://biorender.com).

The circadian rhythm plays an important role in glucose metabolism by sustaining daily fluctuations in physiological, hormonal, and behavioral processes [3,81]. Especially, the central circadian clock in the SCN is a pacemaker which aligns our daily sleep-wake, activity-rest as well as feeding-fasting cycles and synchronizes the metabolic activity of peripheral clocks [82–85]. Therefore, the central and peripheral circadian clocks jointly regulate endogenous diurnal rhythm in glucose metabolism (Figure 2). The sleep-wake cycle is the main manifestation of the endogenous circadian clock and is also the metabolic master switch [81]. A normal sleep-wake pattern aligned with the circadian rhythm supports normal metabolism; for example, energy expenditure during sleep is just two-thirds of that during wakefulness [81,86]. Moreover, the rhythm of the sleep-wake cycle corresponds to the fasting and feeding rhythm and thereby influences glucose metabolism synergistically [81]. The glucose tolerance in response to the same meal is relatively impaired in the evening/night compared with the morning in healthy individuals [4]. Thus, glucose metabolism oscillates with the circadian rhythm such that decreased glucose tolerance and a lower response of β-cells occur at night. The progressive impairment of glucose tolerance and insulin sensitivity due to long-term shift work or circadian misalignment might be due to the joint action of a destroyed endogenous rhythm and altered feeding pattern [87].

Several hormones affecting the fluctuation of glucose tolerance and insulin secretion, such as corticosteroids, melatonin and growth hormone, were also found to be controlled by the daily rhythm [88]. Growth hormone is an anabolic hormone with levels that increase during the night and peak immediately after sleep onset [88]; this hormone can rapidly elevate glucose levels by inhibiting muscle glucose uptake and suppressing glucose oxidation [89]. Cortisol release depends on the interaction between the SCN and the PVN. The nadir of human endogenous cortisol occurs at midnight, and the levels rise for 2-3 h after sleep onset, followed an increasing trend until 9 o’clock in the morning (the peak); a lower level is then maintained during the day, with a decline usually during the later afternoon [90]. Cortisol plays a critical role in the wakening response in the early morning and in glucose homeostasis. Circadian disruption disturbs the daily rhythm of cortisol secretion and induces hypercortisolemia, which leads to hepatic and peripheral insulin resistance [91].

### 3.2. Melatonin as the “circadian zeitgeber” for glucose metabolism

**Figure 3.**
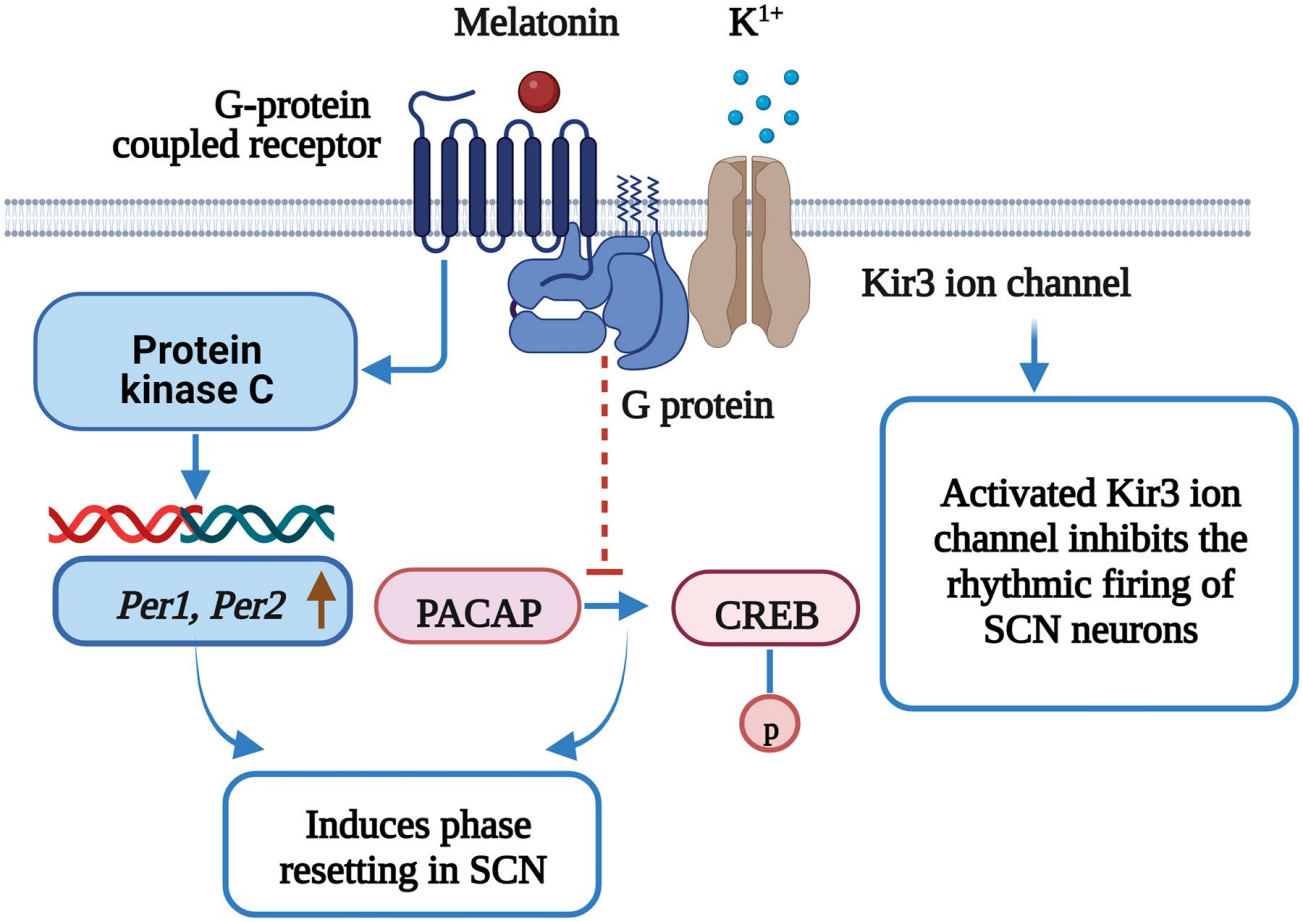
Melatonin acts as a circadian pacemaker and advances the SCN phase [93–97]. The retinohypothalamic tract mediates cAMP responsive element binding protein (CREB) phosphorylation via pituitary adenylate cyclase-activating polypeptide (PACAP) release under light stimulation in SCN cells; PACAP release is responsible for light-induced phase shifts. The binding of melatonin to MT1 inhibits PACAP-induced CREB phosphorylation in the SCN. Melatonin receptors activate G-protein-coupled Kir3 ion channels, inhibit the rhythmic firing of SCN neurons and regulate circadian rhythms. Melatonin activates PKC in the SCN and induces phase resetting, and through this signaling, the expression of core clock genes, *Period 1* (*Per1*) and *Period 2* (*Per2*), increases within the SCN. Figure 2 was created with BioRender (https://biorender.com).

Melatonin is a major chronobiotic hormone that provides feedback to the central and peripheral circadian clocks to regulate metabolic function by binding to its receptors (MT1 and MT2), which are expressed in central and peripheral sites, and initiating several specific signal transduction mechanisms. In SCN cells, rhythmic melatonin production inhibits the phosphorylation of cAMP responsive element binding protein (CREB) induced by the retinohypothalamic transmitter pituitary adenylate cyclase-activating polypeptide (PACAP); melatonin production thus regulates neuronal sensitivity to endogenous clock phase shifts [92] (Figure 3). *In vitro* studies have shown that melatonin receptor signaling increases the activity of Kir3 ion channels (G-protein-coupled potassium channels) in the SCN and hypothalamus, which inhibits the firing rate of neurons and the regulation of the circadian system [93]. Melatonin can also facilitate circadian clock resetting by activating the protein kinase C (PKC) pathway *via* MT2 signaling [94]. Intriguingly, melatonin acts as a circadian modulator that alters the expression of clock genes (*per1*, *per2*, *cry1*, *bmal1* and *clock*) *via* PKC signaling in the SCN [95–97], which entrains neuronal activity rhythms. Melatonin, as the main signaling molecule of the central circadian clock, can coordinate human physiological metabolism and behavioral activities with the circadian temporal structure.

**Figure 4.**
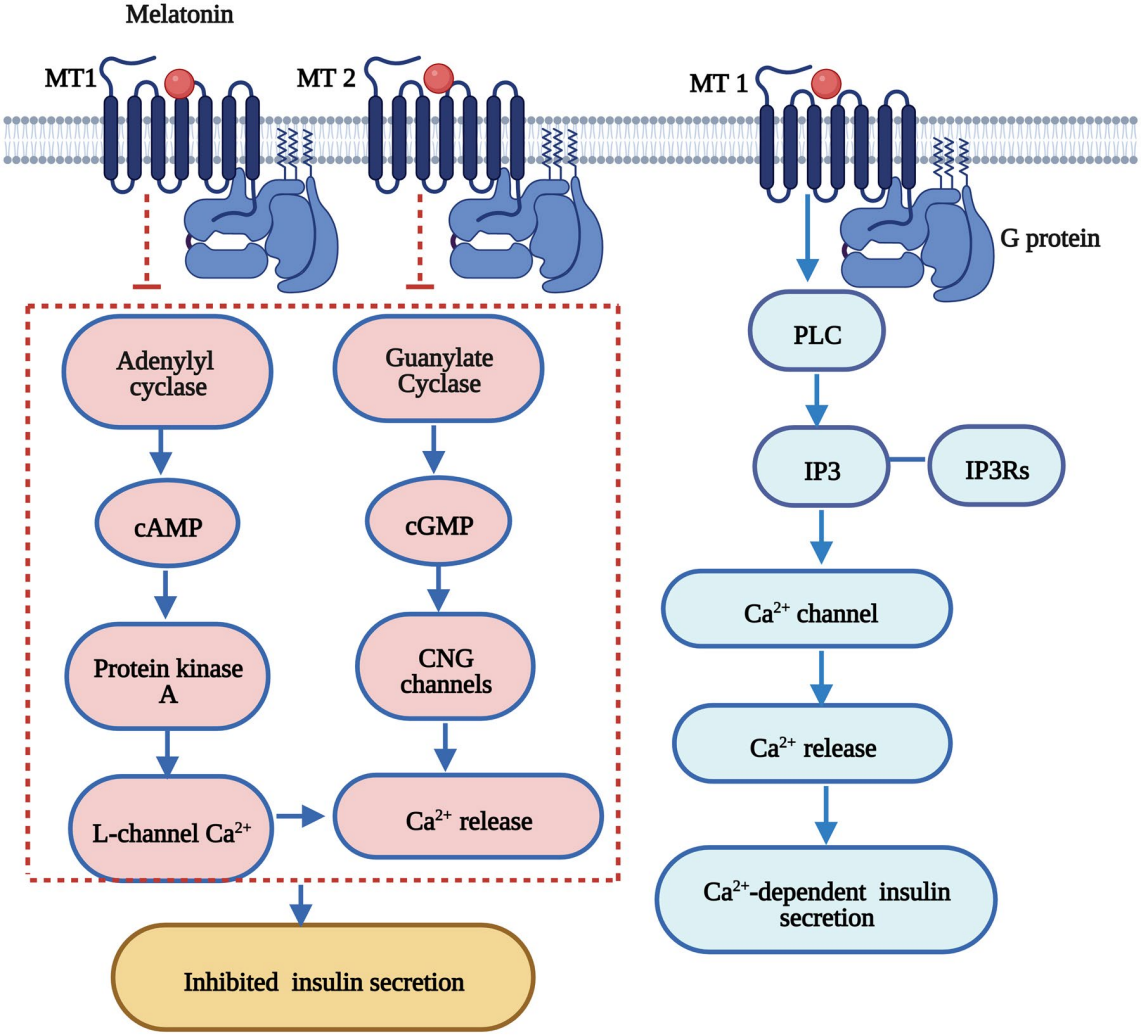
Melatonin regulates insulin secretion in pancreatic β-cells *via* the cAMP, cGMP and IP3 signaling pathways [98,236]. The binding of melatonin to MT1 inhibits cyclic adenosine monophosphate (cAMP) signaling and decreases insulin secretion in pancreatic β-cells. The binding of melatonin to MT2 inhibits cyclic guanosine monophosphate (cGMP) signaling and decreases insulin secretion in pancreatic β-cells. Melatonin stimulates IP3 release accompanied by a transient increase in Ca^2+^ concentrations and leads to Ca^2+^-dependent insulin secretion. Figure 3 was created with BioRender (https://biorender.com).

**Figure 5.**
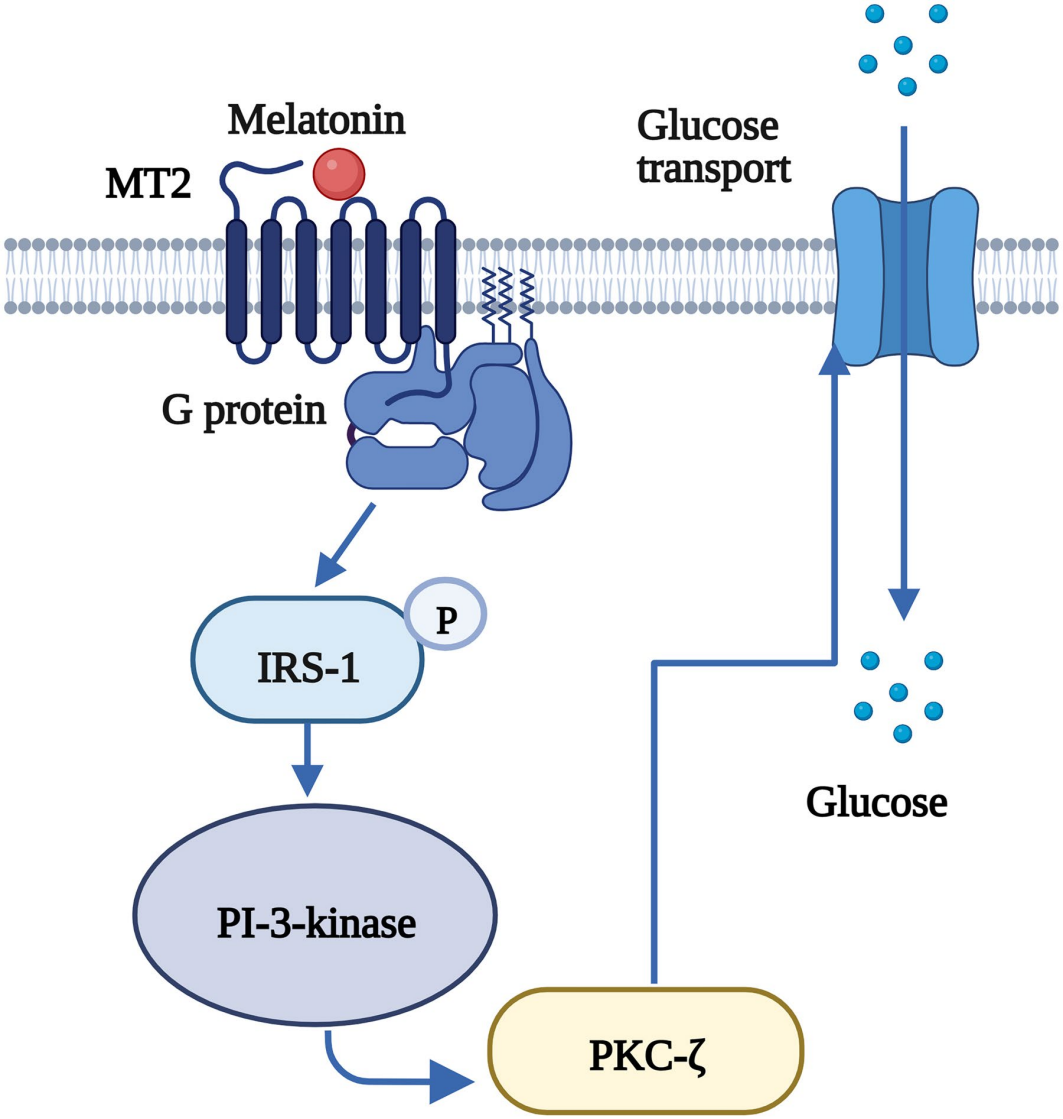
The binding of melatonin to MT2 stimulates glucose transport to skeletal muscle cells *via* the IRS-1/PI-3-kinase pathway [101]. Melatonin increases the phosphorylation level of insulin receptor substrate-1 (IRS-1) and the activity of phosphoinositide 3-kinase (PI-3-kinase), activates downstream protein kinase C (PKC)-ζ and stimulates glucose transport to muscle cells. Figure 4 was created with BioRender (https://biorender.com).

**Figure 6.**
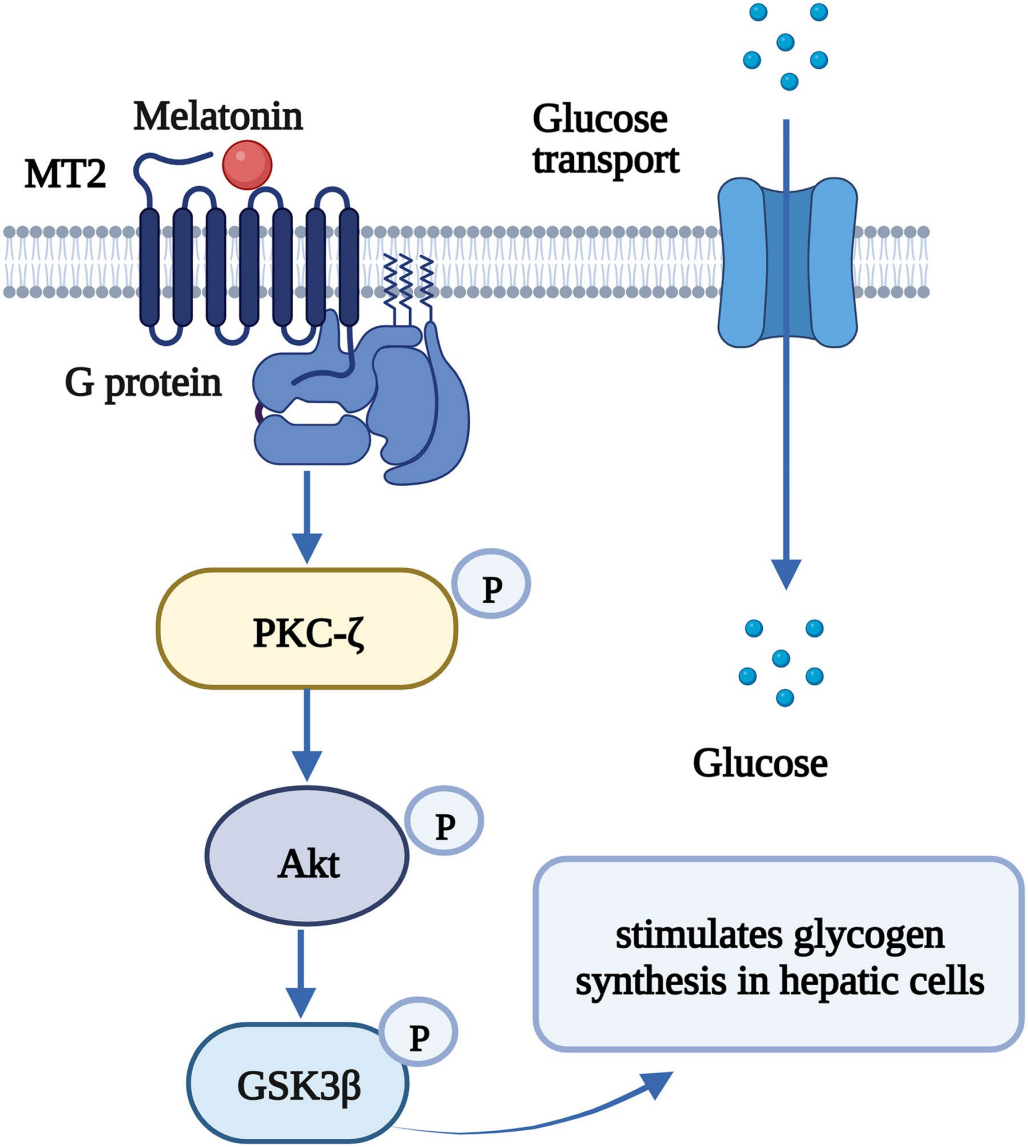
The binding of melatonin to MT1 stimulates glycogen synthesis in hepatic cells *via* the protein kinase Cζ (PKCζ)-Akt-glycogen synthase kinase 3B (GSK3β) pathway [102]. Melatonin increases the phosphorylation of PKCζ, Akt, and GSK3β and stimulates glycogen synthesis in hepatic cells. Figure 5 was created with BioRender (https://biorender.com).

Furthermore, melatonin receptors are expressed in peripheral tissues and mediate peripheral clocks in the pancreas, muscle, liver, adipose tissue and gut; consequently, these receptors affect glucose homeostasis through various intracellular mechanisms [13,16]. Previous *in vitro* and *in vivo* studies have demonstrated that insulin secretion in pancreatic β-cells is mediated by melatonin *via* cAMP, cyclic guanosine monophosphate (cGMP) and inositol triphosphate (IP3) signaling cascades through the melatonin receptor subtypes MT1 and MT2 [98–100] (Figure 4). Glucose transport to muscle cells was also found to be controlled by melatonin through the activation of insulin receptor substrate-1 (IRS-1)/phosphoinositide 3-kinase (PI-3-kinase) signaling and downstream PKC-ζ *via* MT2 in rats [101] (Figure 5) . Melatonin also has the ability to ameliorate incremental glycogen increases and improve glucose utilization in HepG2 cells through a PKCζ-Akt-glycogen synthase kinase 3 beta (GSK3β)-related pathway *via* melatonin-specific receptors [102] (Figure 6). Hence, melatonin signaling is a potent ‘internal zeitgeber’ that entrains the endogenous circadian rhythm and synchronizes various organs and tissues to control glucose metabolism.

Given the critical role of melatonin in glucose homeostasis, the effect of melatonin signaling on T2DM has been widely studied. Rats lacking melatonin showed marked glucose intolerance and insulin resistance, which may result from a widespread reduction in glucose transporter-4 (GLUT-4) in many insulin-targeted adipose tissues, such as adipose and hepatic cells [103,104]. Rats with experimentally induced diabetes displayed lower peak melatonin levels, pancreatic melatonin receptor overexpression and higher insulin levels during glucose metabolism impairment [105,106]. In diabetic rats, combined treatment with low-dose melatonin and insulin ameliorated insulin sensitivity in adipose tissue and improved glycemic control [107], supporting a dynamic interaction between melatonin and insulin signaling [107]. In insulin-resistant mice, the administration of melatonin enhanced insulin function related to vasodilatation, which facilitated glucose transport in skeletal muscle and subsequently improved glucose utilization [108]. Population-based studies have also reported the beneficial effect of melatonin regarding glucose homeostasis. Case–control and prospective cohort studies confirmed a higher risk of glucose tolerance, insulin sensitivity and T2DM in participants with decreased melatonin [19,23,109]; treatment with melatonin elevated the levels of adiponectin, leptin, and ghrelin; reduced insulin resistance; and improved glycemic traits and diabetic complications [5,110,111]. Although the studies above provide evidence of the beneficial role of melatonin in the circadian rhythm and glycemic control, recent studies have only partially elucidated the causal relationship between melatonin signaling and T2DM risk. It is possible that genetic effects may also be critical in the development of T2DM.

## 4. *MTNR1B* gene and T2DM

### 4.1. Genetic variants of MTNR1B affect glucose traits and T2DM

The physiological functions of melatonin are mainly mediated by the specific high-affinity receptors MT1 and MT2. *MTNR1B* encodes melatonin receptor MT2, which is expressed in the human brain, pancreatic islets and β-cells [28], and is a prime candidate gene for glycemic and metabolic traits, especially the common variant rs10830963 within the single 11.5-kb intron of *MTNR1B*. A previous meta-analysis combined data from 10 GWASs of individuals of European ancestry free of diabetes that validated the appreciable associations between several variant loci (rs1387153, rs11020107 and rs10830963) of *MTNR1B* and fasting glucose levels as well as reduced β-cell function [28]. The strongest signal consistently occurred at rs10830963, and the fasting glucose concentration was elevated by an average of 0.07 mmol/l per risk (G) allele [28]. Moreover, the findings from another extensive meta-analysis including 21 GWASs of 46,186 nondiabetic participants of European descent were consistent with the aforementioned evidence and indicate the effects of *MTNR1B* rs10830963 on fasting glucose and β-cell function [30]. Subsequent replication studies in large-scale case–control and cohort research provided robust evidence that the glucose-elevating allele (*MTNR1B r*s10830963) was associated with glycemic traits such as impaired glucose tolerance, inhibited β-cell function, increased fasting glucose levels and T2DM in Europeans [30,112–114], Asians [115–118], North Americans [119] and African Americans, among others [120,121]. In addition, GWASs in different populations, including lean adults, lean adolescents, obese adults and obese adolescents, as well as subsequent replication analyses all identified that rs1387153 (in the 5′ region of *MTNR1B*) T allele was linked with elevated concentrations of fasting plasma glucose and glycosylated hemoglobin (HbA1c) as well as elevated T2DM risk [28,113]. Furthermore, a similar study confirmed that rs10830963 was in linkage disequilibrium (LD) with rs1387153, supporting the strongest genetic role of rs10830963 at *MTNR1B* in T2DM [29,112]. Table 2 summarizes the GWASs investigating glycemic traits and T2DM since 2008 [28–30, 113,114,119,122–170].

**Table 2.** Common variants of MTNR1B significantly associated with glycemic traits or T2DM in GWASs.

Publication year	Disease/Trait	Sample size	SNP-Risk allele	OR / BETA	95% CI
2008 [28]	FPG	2151 European ancestry individuals	rs1387153-T	0.07	[0.05–0.08] mmol/l increase
2008 [29]	FPG	35,812 European ancestry individuals	rs10830963-G	0.07	[0.06–0.08] mmol/l increase
2010 [30]	FPG, HOMA	82,652 European ancestry individuals	rs10830963-G	0.07	[0.061–0.073] mmol/L increase
2010 [113]	T2DM	47,117 European ancestry individuals	rs1387153-T	1.09	[1.06–1.11]
2008 [114]	FPG	4763 Northern Finnish founder individuals	rs1447352-G	0.05	[0.03–0.06] mmol/l decrease
2014 [119]	Glucose homeostasis traits	4176 Mexican American individuals	rs10830963-?	2.76	[1.88–3.64] unit decrease
2009 [122]	FPG	74,748 Asian Indian ancestry individuals	rs2166706-G	0.07	(0.04–0.09) mmol/L increase
2010 [123]	HbA1c	46,368 European ancestry individuals	rs1387153-T	0.03	[0.02–0.04] % increase
2011 [124]	GLUC, TG	22,161 European ancestry individuals	rs10830956-C	0.20	[0.14–0.26] unit decrease
2011 [125]	Metabolic traits	12,545 Korean ancestry individuals	rs10830962-C	0.04	[0.034–0.055] mg/dL increase
2012 [126]	FPG	8330 European ancestry individuals	rs10830963-G	0.14	[0.1–0.18] unit increase
2012 [127]	FPG	2,349 European ancestry individuals, 664 Chinese ancestry individuals, 1366 African American individuals, 1171 Hispanic individuals	rs10830963-G	1.38	[1.01–1.75] mg/dL increase
2012 [128]	FPG	58,074 European ancestry individuals	rs10830963-C	0.08	[0.072–0.087] unit decrease
2012 [129]	Obesity-related traits	815 Hispanic children from 263 families	rs10830963-G	0.05	[NA] mg/dL increase
2012 [130]	GDM	1710 Korean ancestry individuals	rs10830962-G	1.45	[1.315–1.608]
2012 [131]	Metabolic syndrome	10,564 European ancestry individuals	rs10830962-G	0.12	[NA] mmol/l increase
2012 [132]	T2DM	69,033 European ancestry individuals	rs10830963-G	1.10	[1.07–1.13]
2013 [133]	FPG	7,696 Korean ancestry individuals	rs10830962-C	0.04	[0.029–0.053] mmol/l increase
2013 [134]	FBG (pregnancy)	1367 European ancestry individuals, 817 Hispanic individuals, 1075 Afro-Caribbean individuals, 1178 Thai ancestry individuals	rs7936247-T	0.22	[NA] unit increase
2014 [135]	Insulin traits	10,651 European and Old Order Amish individuals	rs10830963-G	0.09	[0.054–0.116] unit decrease
2014 [136]	T2DM	17,857 European ancestry individuals, 18,817 East Asian ancestry individuals, 20,019 South Asian ancestry individuals, 2583 Mexican ancestry individuals	rs10830963-G	1.11	[1.06–1.16]
2015 [137]	FPG	50,900 European ancestry non-diabetic individuals, 9664 African American non-diabetic individuals	rs10830963-G	0.07	[0.065–0.079] unit increase
2016 [138]	Metabolite levels	2118 Erasmus Rucphen (founder/genetic isolate), 22,807 European ancestry individuals	rs10466351-T	0.07	[0.05–0.09] unit increase
2016 [139]	Birth weight	133,903 European ancestry individuals, 6635 African American individuals, 420 Turkish ancestry individuals, 365 Moroccan ancestry individuals, 395 Surinamese ancestry individuals, 840 Chinese ancestry individuals.	rs10830963-G	0.02	[0.014–0.03] unit increase
2017 [140]	Peak insulin response, Acute insulin response, Insulin secretion rate, Insulin disposition index	2346 Hispanic individuals, 2159 European ancestry individuals, 332 Pima Indian ancestry individuals, 527 European ancestry individuals	rs10830963-G	0.24	[0.19–0.28] pmol/l decrease
2017 [141]	HbA1c	88,355 European ancestry individuals, 18,472 East Asian ancestry individuals	rs10830963-G	0.02	[0.016–0.024] unit increase
2017 [142]	T2DM	13,532 South Asian ancestry individuals, 20,298 European ancestry individuals, 149,821 other population	rs10830963-G	0.09	[0.071–0.117] unit increase
2017 [143]	T2DM	7746 Hispanic/Latino ancestry individuals	rs10830963-G	1.28	[1.17–1.41]
2018 [144]	Offspring birth weight	68,258 European ancestry women	rs10830963-G	0.05	[0.040–0.064] z–score increase
2018 [145]	HbA1c	42,790 Japanese ancestry individuals	rs10830963-G	0.04	[0.026–0.052] unit increase
2018 [146]	Glycemic traits	5947 European ancestry individuals	rs10830963-G		
2018 [147]	FPG	7647 European ancestry individuals, 2104 Black individuals	rs10830963-G	1.58	[0.97–2.18] unit increase
2018 [148]	T2DM	459,000 European ancestry individuals	rs10830963-G	NA	
2018 [149]	T2DM	6224 African American	rs10830963-G	NA	
2018 [150]	T2DM	127,001 European ancestry individuals, 18,817 East Asian ancestry individuals, 20,019 South Asian ancestry individuals, 2583 Mexican ancestry individuals	rs7113297-T	1.11	
2018 [151]	DN	18,174 European ancestry individuals	rs768920-?	1.39	[1.14–1.69]
2018 [152]	T2DM	655,666 European ancestry individuals, 3650 Pakistani ancestry individuals	rs10830963-G	0.09	[0.075–0.107] unit increase
2018 [153]	T2DM	898,130 European ancestry individuals	rs10830963-G	1.10	[1.09–1.12]
2019 [154]	Glycemic traits	46,186 European ancestry individuals	rs10830963-G	NA	
2019 [155]	Own Birth weight, Offspring birth weight	528,211 European ancestry individuals, 6635 African American individuals, 1449 Filipino individuals, 420 Turkish individuals, 365 Moroccan individuals, 395 Surinamese individuals, 1052 Afro-Caribbean individuals, 612 Hispanic individuals, 1180 Thai individuals, 840 Chinese ancestry individuals, 10,133 individuals	rs10830963-G	0.02	[0.013–0.025] unit increase
2019 [156]	FPG, HbA1c	6457 African American individuals, 13,556 Hispanic/Latino individuals, 1918 Asian ancestry individuals, 1400 Native Hawaiian ancestry individuals, 412 Native American ancestry individuals, 168 individuals	rs10830963-G	0.13	[0.11–0.16] unit increase
2019 [157]	Serum metabolite levels	6263 Finnish ancestry men	rs10830963-G	0.07	NA unit increase
2019 [158]	Waist circumference	457,690 European ancestry individuals	rs10830956-C	NA	
2020 [159]	Insulin-related traits	62,205 individuals	rs11020114	NA	
2020 [160]	FPG	7423 Korean ancestry individuals	rs10830963-G	1.33	[1.233–1.432]
2020 [161]	T2DM	1,114,458 European ancestry individuals, 56,092 African American, 20,445 Hispanic individuals, 216,287 Asian ancestry individuals	rs10830963-G	0.07	[0.065–0.081] unit increase
2020 [162]	T2DM	433,540 East Asian individuals	rs10830963-G	1.04	[1.02–1.05]
2021 [163]	FPG	151,188 European ancestry individuals	rs10830963-C	0.08	[0.072–0.082] unit decrease
2021 [164]	HbA1c	327,177 European ancestry individuals, 4847 African ancestry individuals, 6895 South Asian ancestry individuals	rs1387153-T	0.05	[0.047–0.058] unit increase
2021 [165]	FPG	18,122 Han Chinese ancestry individuals	rs10830963-G	0.11	[0.085–0.125] unit increase
2021 [166]	HbA1c, FPG	200,622 European ancestry individuals, 35,619 East Asian ancestry individuals, 16,579 African American individuals, 19,247 Hispanic individuals, 9343 South Asian ancestry individuals, 33,307 East Asian ancestry individuals	rs10830963-C	0.02	[0.017–0.023] unit decrease
2021 [167]	HbA1c, T2DM	628,000 European ancestry individuals, 179,000 East Asian ancestry individuals	rs10830963-G	0.05	[0.048–0.057] unit increase
2021 [168]	Glucose meal response, FPG	11,410 individuals	rs10830963-G	0.23	[0.18–0.28] unit decrease
2021 [169]	FPG	19,745 European ancestry individuals, 12,195 African American/Afro-Caribbean individuals, 16,018 Hispanic/Latin American individuals	rs10830963-C	0.16	[0.13–0.18] unit decrease
2022 [170]	FG, FI	23,000 individuals from five race ethnicities (African, Asian, European, Hispanic and Samoan)	rs10830963-G	0.07	[0.05–0.09] unit increase

DN: diabetic nephropathy; FPG: fasting plasma glucose; GDM: gestational diabetes mellitus; T2DM, type 2 diabetes mellitus; GLUC, glucose; HbA1c: hemoglobin A1c; HOMA: homeostatic model assessment; NA; not available.

### 4.2. MTNR1B is associated with therapeutic and intervention targets for T2DM

A study also reported that T2DM patients with the *MTNR1B* rs10830963 G risk allele experienced attenuated therapeutic efficacy of hypoglycemic treatment, such as repaglinide and nateglinide [171,172]. This attenuation is possibly due to the increased expression of *MTNR1B* in pancreatic β-cells; *MTNR1B* has an antagonistic effect on the triggering of insulin release by antidiabetic pharmacotherapy. This finding also provides a personalized therapy target for specific diabetes patients. Another study found that a high rate of insulin treatment efficacy could not be predicted in rs10830963 G risk allele carriers with T2DM [173]. Remarkably, in women with normal glycemic levels, the *MTNR1B* rs10830963 risk variant predicted approximately 26% of individual variation in the curative effect of melatonin on glucose tolerance [173]. In healthy participants, after the administration of 5 mg of melatonin in the morning (9:00 am), the deleterious effect of melatonin on glucose tolerance was elevated six-fold in *MTNR1B* G allele carriers compared with noncarriers [174]. Another clinical trial found that in healthy participants, the administration of a supraphysiological dose of melatonin led to reduced insulin sensitivity in homozygous rs10830963 CC carriers [175]. Therefore, we suspect that *MTNR1B* acts as a genetic bridge between circadian rhythms and glucose metabolism, and the common variant (rs10830963) could provide predictive biomarkers and therapeutic targets for T2DM.

Curiously, a large-scale population-based study reported an inverse correlation of the risk allele of *MTNR1B* with the hepatic insulin resistance (IR) index [113] in nondiabetic participants. IR in the liver generally contributes to impaired hepatic glucose synthesis and enhanced gluconeogenesis and glycogenolysis, ultimately leading to hyperglycemia or T2DM [176,177]. Researchers hypothesized that the increased hepatic insulin sensitivity of *MTNR1B* risk allele carriers might be a compensatory mechanism that balances impaired insulin secretion and the maintenance of a normal range of glycemia levels in nondiabetic participants [178,179]. Studies have consistently demonstrated that *MTNR1B* is a true causal gene for diabetes, but the underlying molecular mechanisms remain unclear.

Functional data has shown that individuals with the risk genotype have higher expression of *MTNR1B* in human pancreatic β-cells and that *MTNR1B* expression is positively correlated with impaired insulin secretion [18,180,181]. As previously mentioned, melatonin signaling modulates insulin gene expression *via* MT2, and *MTNR1B* overexpression aggravates the inhibitory effect of melatonin on the insulin gene; however, silencing *MTNR1B* diminishes the above effect in pancreatic β-cells. Thus, melatonin-MT2 binding may inactivate the mitogen-activated protein kinase (MAPK) pathway and eventually reduce insulin gene expression in β-cells [182]. Genomic annotation and functional assays in individuals of European ancestry have indicated that the *MTNR1B* variant region overlaps with the forkhead box transcription factor A2 (*FOXA2*)-bound site and subsequently reinforces *FOXA2*-bound enhancement of activity in human islet- or liver-derived cells [183]. The risk allele of rs10830963 also has a specific binding site that matches with *NEUROD1* in human islet cells [183]. Thus, the common variants of rs10830963 drive signaling by inducing islet *MTNR1B* expression by enhancing *FOXA2* expression and binding to *NEUROD1*. However, large-scale exon sequencing revealed 40 nonsynonymous *MTNR1B* variants and four very rare variants (with an MAF < 0.1%), which contributed to the total deficits in MT2 function and T2DM risk [184]. The findings from this research suggest that the increased expression of *MTNR1B* was not the cause of T2DM but rather a phenomenon occurring under reduced negative feedback due to the *MTNR1B* mutation and impaired G protein signaling; in other words, the increased expression of *MTNR1B* was a compensatory and adaptive activation *via* intracellular regulatory mechanisms. The attenuated effect of the melatonin system on insulin release regulation and circadian rhythms appeared to be a key cause of T2DM. Overall, more evidence is needed to clarify the effects of *MTNR1B* variants on the risk of T2DM and to provide definite therapeutic targets for the disease.

*MTNR1B* is also expressed in other human islet cell types, including α- and δ-cells, which determine the stimulatory impact of melatonin on glucagon and somatostatin secretion, respectively [100,185]. Physiological concentrations of melatonin elevate somatostatin and decrease somatostatin release both *in vitro* and *in vivo*; these two hormones are important regulators of blood glucose homeostasis [186,187]. However, *MTNR1B* density is lower in human pancreatic islet α-cells than in β-cells, and *MTNR1A* is the major transmitter of the inhibitory effect of melatonin in human δ-cells [185]. Scant evidence on the influence of genetic variants of *MTNR1B* on the function of α-cells in humans is available; however, Anna Jonsso et al. found that the strongest correlation between glucose levels and glucagon occurred in individuals with the *MTNR1B* rs10830963 variant [188]. Future studies are needed to determine whether *MTNR1B* variants impair insulin secretion and sensitivity in pancreatic islet β-cells and promote glucagon release in α-cells.

### 4.3. MTNR1B contributes to the common genetic background for GDM and T2DM

Many studies have assumed that gestational diabetes mellitus (GDM) shares genetic roots with T2DM, and women with GDM may have 8.9-folds increased risk of T2DM [189]. A multiancestry genome-wide association study found four risk loci (*MTNR1B*, *TCF7L2*, *CDKAL1*, and *CDKN2A-CDKN2B*) for GDM, which were all linked with the risk of T2DM [190]; these findings also indicate that GDM and T2DM have shared pathophysiology. Three variants of *MTNR1B* (rs10830963, rs1387153 and 10830962) exhibited genome-wide significant associations with GDM [191]. The *MTNR1B* risk allele of rs10830963 was also associated with alterations in gestational glucose tolerance, including impaired insulin sensitivity, increased early-phase insulin release and fasting insulin conversion in women without GDM [192]. Similarly, the *MTNR1B* rs7936247 T allele was strongly associated with elevated fasting plasma glucose (FPG) levels in pregnant women [134]. Moreover, another study also showed that *MTNR1B* rs10830962 and rs1387153 were genetic risk loci for GDM in both Asian and Caucasian cohorts [130,193–196].

Common variants of *MTNR1B* are linked to substantial heterogeneity in glycemic control during pregnancy. Among pregnant women with prior GDM, the effectiveness of lifestyle intervention was attenuated by the rs10830963 risk allele (G); only women homozygous for the rs10830963 C allele benefitted from the lifestyle intervention and showed a decrease in GDM risk [197]. The *MTNR1B* rs10830962 G allele also increased the risk of GDM, and in women homozygous for the rs10830962 G allele, physical activity decreased maternal fasting insulin and insulin resistance (according to the homeostatic model assessment of insulin resistance, HOMA-IR) [198]. Thus, it seems that genetic susceptibility loci of *MTNR1B* could be candidate pharmacogenetic markers and prevention targets for GDM in risk population subgroups. Recent evidence further suggested that the maternal G allele at *MTNR1B* rs10830962 increased the risk of childhood obesity and metabolic abnormalities in offspring given observed interactions with gestational weight gain in a GDM mother-child paired cohort [199]. A cohort study followed women with a GDM history during postpartum years 1-5; the results showed that women with the *MTNR1B* rs10830963 GG genotype had higher postpartum fasting glucose levels [200]. Thus, *MTNR1B* may be part of a common genetic predisposition for GDM and T2DM.

**Figure 7.**
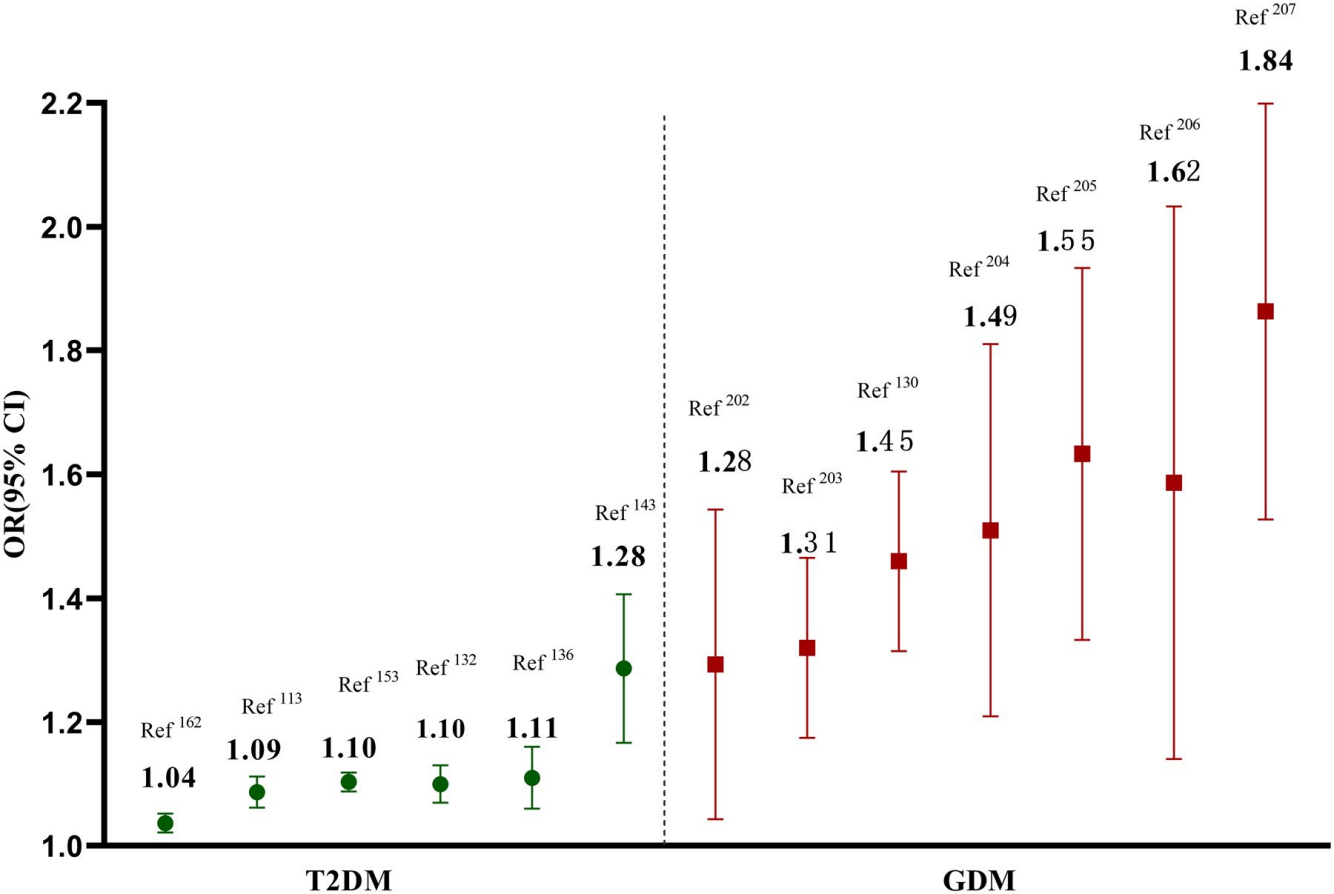
The different genetic effect sizes of *MTNR1B* in T2DM and GDM.

Although, the common variants of *MTNR1B* provided a shared genetic basis between GDM and T2DM, but the genetic effect sizes were pronounced difference [201]. Especially, the *MTNR1B* rs10830963 presented a higher genetic effect size for GDM when compared to T2DM, and the odds ratios (*ORs*) of rs10830963 risk allele were generally ranged from 1.04 to 1.28 for T2DM, but from1.28 to1.84 for GDM [113,132,136,143,153,162,202–207] (Figure 7). Pregnancy is a special physiological state which is accompanied by progressive insulin resistance, but just a small proportion of pregnancy women developed as GDM. Previous study had reported increased nighttime serum melatonin levels after 24 weeks of gestation [208], and this hormone could aggravate insulin desensitization. Concurrently, the expression levels of *MTNR1B* in placenta seem to up-regulate among GDM women, especially in participants with rs10830963 GG and GC genotypes [209]. The pregnant women of concurrent rising levels of melatonin and *MTNR1B* gene contribute to increased insulin resistance, which may be genetically predisposed to GDM. Moreover, several specific environmental factors altered during pregnancy, such as maternal adiposity, placenta hormones and paternal or fetal genotypes, may modulate the genetic effects of *MTNR1B* on maternal GDM *via* gene-environment or gene-gene interactions [202].

### 4.4. The different genetic effect sizes of MTNR1B in Sub-populations

Notably, several other factors, including body mass index (BMI), age and gender should be taken into account when assessed the genetic effect of *MTNR1B* on glycemic traits. The genetic effects of *MTNR1B* on glycemic traits appeared to be more preponderant in overweight and obese individuals [203,210–214] (Table 3). A meta-analysis detected the effects of an interaction between the *MTNR1B* rs10830963 genetic variant and prepregnancy BMI on the risk of GDM; specifically, rs10830963 G carriers with a pre-pregnancy BMI ≥25 kg/m^2^ were more susceptible to GDM [203]. Among GDM patients with a prepregnancy BMI ≥29 kg/m^2^, carriers of the *MTNR1B* rs10830963 G risk allele presented a five-fold higher rate of antenatal insulin therapy (AIT) initiation [214]. Studies with overweight/obese children and adolescents also confirmed an allele-dosage effect of the rs10830963 G allele on impaired fasting glucose and B-cell functions [210,212,213]. Furthermore, several *MTNR1B* variants, such as rs76371840, rs8192552, rs6177139, rs6483208, rs4388843, rs4601728, and rs12804291, are associated with obesity traits, and may also increase the risk of T2DM indirectly [171]. Therefore, the interaction between *MTNR1B* and BMI could provide critical candidate targets for glucose metabolism.

**Table 3.** The different genetic effects of *MTNR1B* on glycemic traits in overweight/obesity individuals.

Reference	Study population	Risk allele	subgroup	B-estimate (SEM)/OR (95% CI)
^[210]^	310 children and adolescents, aged 8-19 years, Caucasian populations.	rs10830963-G	BMI-SDS < 2.5	Reference
			2.5 ≤ BMI-SDS < 3.0	0.209 (0.093) mmol/L increase of FBG 0.144 (0.178) decrease of HOMA–B
			BMI-SDS ≥ 3.0	0.265 (0.091) mmol/L increase of FBG 0.380 (0.174) decrease of HOMA–B
^[211]^	1002 adult obese subjects, aged 26–66 years, Caucasian populations.	rs10830963-G		1.31 (1.12–2.78) increase the odds of hyperglycemia 1.37 (1.14–2.86) increase the odds of diabetes mellitus
^[212]^	781 obese children and adolescents, mean aged 13.4 ± 3.6 year, 346 Caucasians, 218 African–Americans, 217 Hispanics.	rs10830963-G	African–Americans Hispanics	3.72 (1.36–10.12) increase the odds of IFG 2.50 (1.01–6.18) increase the odds of IFG
			Caucasians	3.12 (1.41–6.88) increase the odds of IFG and IGT
			African–Americans	3.29 (1.203–8.98) increase the odds of risk of IGT
^[213]^	1,118 overage children and adolescents, aged 6–16 years, Caucasian populations.	rs10830963-G		1.101 (0.316—1.886) mg/dL increase of FBG
^[203]^	2772 pregnant women	rs10830963-G	Pre-pregnancy BMI <25	1.22 (0.9, 1.65)
			Pre-pregnancy BMI ≥25	1.24 (1.02–1.51) increase the odds of GDM
^[214]^	211 GDM patients Caucasian populations	rs10830963-G	Pre-pregnancy BMI <29	1.36(NA)
			Pre-pregnancy BMI ≥29	5.2(1.3–20.8) increase the odds of

BMI-SDS: body mass index standard deviation score; HOMA-B: homeostasis model assessment of β-cell function; AIT: antenatal insulin therapy; FBG: fasting blood glucose; IFG: impaired fasting glucose; IGT: impaired glucose tolerance; GDM: gestational diabetes.

Cohort studies indicated that *MTNR1B* rs10830963 was associated with elevated fasting glucose in early life among nondiabetic individuals [112,180]. The common genetic variants contributed to a stable age-related rise of fasting glucose, but a more pronounced age-related increase of 2-h postload glucose [180,215]. These results were consisted with the impaired insulin secretion in carriers with risk *MTNR1B* genotype over the lifespan [180]. Another longitudinal observation indicated that the *MTNR1B* rs10830963 risk allele led to a faster rate of progression from normal fasting glucose to impaired fasting glucose (IFG) than the rate of progression from IFG to T2DM [216]. Therefore, age may modify the heterogenetic effects of *MTNR1B* risk alleles on glycaemic phenotype *via* progressively impaired fasting glucose and insulin response

Gender differences in the genetic effects of *MTNR1B* on T2DM remain elusive. A population-based analysis presented similar trajectories of glucose, insulin and C-peptide in rs10830963 CC, CG, and GG genotypes among young healthy volunteers, but the glycemic curves exhibited gender differences [217]. Specifically, the glycemic curves were monophasic, biphasic, triphasic, or more complex in healthy volunteers. The proportion of biphasic glycemic curves in men was twice as high as that in women, while the ratio of triphasic glycemic curves was significantly higher in women than in men [217]. A joint analysis of nine diabetes-related genes (*MTNR1B, TCF7L2, KCNJ11, HHEX, SLC30A8, WFS1, KCNQ1, FTO,* and *PPARG*) indicated that the weighted risk score of these diabetes risk alleles predicted impaired glucose tolerance (IGT) in females after adjustment for age, BMI and insulin sensitivity; no such association was found in males [218]. However, another large cohort study of 5,327 nondiabetic men found that 8 T2DM-related genes (*MTNR1B*, *TCF7L2*, *KCNJ11*, *HHEX*, *SLC30A8*, *CDKN2B*, *CDKAL1*, and *IGF2BP2*) were associated with impaired early-phase insulin release, individually or in combination [219]. A randomized crossover study in healthy men aged 20-40 years reported that the effects of acute-term melatonin treatment on glucose metabolism were modified by *MTNR1B* genotypes and that deteriorated insulin sensitivity seemed to be driven by the *MTNR1B* rs10830963 C allele; however, the glucose levels were unchanged on melatonin days compared to placebo days [175]. A similar clinical trial in young women (mean age: 24 ± 6 years) confirmed a melatonin-induced impairment of glucose tolerance in carriers of the *MTNR1B* rs10830963 G allele [174]. The complicated interactions between *MTNR1B* and gender and their effects on circadian rhythm and T2DM have yet to be fully elucidated.

### 4.5. Is MTNR1B the genetic overlap of T1DM and T2DM?

T2DM and type 1 diabetes mellitus (T1DM) are etiologically different diabetes subtypes. Genetic overlap between the two forms of diabetes mellitus has rarely been reported. A previous study in children found no association of the T2DM susceptibility gene *MTNR1B* with islet autoimmunity and T1DM [220]. However, a Finnish cohort study found that carriers of the *MTNR1B* rs10830963 G allele were predisposed to T1DM and latent autoimmune diabetes in adulthood (LADA) among participants older than 35 years [221]. Another study used data-driven cluster analysis to divide individuals with new-onset diabetes into five subgroups; they found that rs10830963 was associated with severe autoimmune diabetes (SAID), which is characterized by relatively low BMI, insulin deficiency, metabolic disturbance and positivity for glutamic acid decarboxylase antibodies (GADAs) [222]. However, the available data are still limited to clarifying the role of *MTNR1B* in T1DM.

## 5. Evolutionary selection pressures on *MTNR1B* in T2DM

T2DM is a complex metabolic disease with high heritability. The common variants of *MTNR1B* were unequivocally classified as genetic determinants of energy expenditure and metabolic abnormalities, including glycemic and adiposity traits, which are implicated in T2DM [140]. Recent evolutionary evidence suggests positive genetic selection at *MTNR1B* for energy expenditure, glucose regulation and the circadian rhythm, which may make *MTNR1B* a thrifty gene. The “thrifty gene hypothesis” may partially resolve the evolutionary puzzle of *MTNR1B* and provide a potential explanation for T2DM.

### 5.1. The thrifty gene hypothesis and T2DM

The thrifty genotype of T2DM was first proposed by Neel in 1962; he speculated that thrifty genes promoted survival advantage during periods of famine *via* enhanced energy storage, fat deposition and glucose regulation [223]. However, in modern times with plentiful food supply, the thrifty gene for efficient intake and utilization of food favors the occurrence of T2DM. Subsequent research even distinguished “thrifty early” and “thrifty late” genes [224]. Human ancestors exhibited a hunter-gatherer lifestyle with feast and famine during the day and night, respectively. This lifestyle did not change until the arrival of the Neolithic Age approximately 10 thousand years ago, when humans began to domesticate animals and grow plant crops as food sources. Since then, humans have had a regular diet, although with insufficient nutrients; this regular diet gradually became the template of diet habits in modern society. The “thrifty early” gene hypothesis assumes that the coalescence of autosomes of humans was completed approximately 1 to 2 million years ago; thus, the genetic variants (thrifty alleles) occurring in ancestral genes before 2 million years ago were shared in the whole population. This perspective seems to be consistent with Neel’s assumption [224]. “Thrifty late” genes are theorized to have arisen much later in human evolution, after the differentiation of living environments and evolutionary selection after the out-of-Africa migration [224,225].

The thrifty gene hypothesis has sparked great interest in genetic risks for T2D among different ethnic groups over the years. Previous studies provided genetic evidence for the role of the transcription factor 7-like 2 (*TCF7L2*) gene in the relationship between T2DM and adaptive evolution [226,227]; the researchers observed potential positive selection for HapA (a cluster of homogeneous haplotypes in *TCF7L2*), which was linked with T2DM, as well as several phenotypic traits related to energy metabolism among East Asian, European and West African populations [227]. Another study in 2009 found high population differentiation for rs7901695 at *TCF7L2* but failed to acquire a general signal of positive selection for 17 obesity and T2DM risk loci [228]. In addition, 58 T2DM- or obesity-associated loci from GWASs were examined in 53 different populations by geographical region; the findings implied that different populations experienced substantial variation in selection pressures on obesity and T2DM risk alleles, and no evidence was able to distinguish the ancestral or derived thirty genotypes for T2DM/obesity [229]. The specific variants tended to appear in specific populations; for example, the region containing T2DM loci exhibited predominantly group-specific differentiation between East Asians and Sub-Saharan Africans [229]. A large genetic analysis of thrifty genes in 65 T2DM index SNPs failed to identify a global signal for positive selection but detected several positively selected loci in one or more specific populations; for example, five SNPs in *PROX1*, *GRB14*, *UEB2E2*, *IGF2BP2* and *ARAP1* in African populations; three SNPs in *PROX1*, *HMGA2* and *PRC1* in European populations; and nine SNPs in *NOTCH2*, *THADA*, *GRB14*, *WFS1*, *TP53INP1*, *TCF7L2* and *PRC1* in East Asian populations [224]. Some studies have provided a potential explanation for this phenomenon: the extreme disparities in T2DM risk allele frequencies across diverse populations from Sub-Saharan Africa and from Europe to East Asia may have been caused by an adaptation to different climates, agricultural revolutions or dietary components after human mass migration [230,231]. Neel also emphasized the influences of the complex interplay of genetic and environmental factors on T2DM 36 years after proposing the thrifty gene concept; he supported the role of detrimental lifestyle changes in industrialized societies in genetic homeostasis among populations at high risk of T2DM [232].

### 5.2. Selective pressure on MTNR1B is linked with T2DM

Early evolutionary studies of the *MTNR1B* gene implied positive selection on the lineage leading to human adaptation [37,38]. Human G-protein-coupled MTNR1B consists of 362 amino acids, and its predicted membrane topology comprises four extracellular domains, seven transmembrane domains, four cytoplasmic domains, an extracellular N-terminal domain and an intracellular C-terminal domain [233]. The *MTNR1B* gene is on chromosome 11q21-22 and has two exons separated by an intron [234]. It is encoded by the first exon from the N-terminal extracellular domain to the first intracellular loop; the other six transmembrane domains and C-terminal domain are coded by the second exon [234]. Phylogenetic analysis revealed a positional difference in the evolutionary rate of the MTNR1B amino acid sequence. For instance, the transmembrane domains were relatively conserved, while the N-terminal domain and an intracellular C-terminal domain have several positively selected sites. Moreover, in primates, an additional 4 sites of positive selection were specifically detected in extracellular domains (2 sites), cytoplasmic domains (1 site) and the intracellular C-terminal domain (1 site) [234].

Population genetic analyses based on HapMap SNP data suggest evolutionary selective pressure on *MTNR1B* alleles [37]. Several reported variant loci in *MTNR1B*, such as rs10830963, rs1280429 and rs7951037, were mapped in introns, but rs10830962 and rs4753426 were mapped in the 5′ region. The *MTNR1B* rs1830963 G allele and rs4753426 C allele, which have a detrimental impact on β-cell function as well as glucose levels, exhibited ethnic diversity of risk allele frequencies between Central Europeans and Asians (Chinese Han population and Japanese population) [37]. The frequency of the rs10830963 G allele was also higher in Europe compared to Africa [235]. The different risk allele frequencies of *MTNR1B* loci may be due to a combination of a genetic drift effect and evolving adaptation. The *MTNR1B* variants generated from evolutionary pressures may act as intermediates that link the circadian rhythm with glucose homeostasis. *MTNR1B* rs10830963 G carriers had a higher risk of T2DM due to its mediation of melatonin secretion, the circadian rhythm and other physiological activities [32]. The risk allele (rs1830963 G) was associated with later melatonin secretion onset in the dim-light phase as well as prolongation of these elevated melatonin levels to the morning; risk allele carriers who woke up earlier were at higher risk of T2DM [32]. Perhaps the common variant at rs1830963 helped human ancestors adapt to a lack of food intake during the long night and even in the morning during the hunter-gatherer stage, and the delayed onset and prolonged duration of melatonin secretion prevented nocturnal and early-morning hypoglycemia. However, in modern society, carriers of this genetic variant maintain a relatively elevated fasting glucose level as well as increased insulin resistance from night to morning, which especially aggravates glucose tolerance in the morning after a substantial breakfast. Hence, we hypothesize that in individuals with the rs1830963 risk allele who wake up early in the morning, the effects of the risk variants on T2DM risk, such as impaired fasting glucose and glucose tolerance, are magnified. Moreover, the frequency of the *MTNR1B* rs4753426 C allele was negatively correlated with environmental sunshine duration among populations originating from different geographic regions [38], which indicates that this disease-predisposition gene in the melatonin system is a candidate gene subject to ambient light and circadian rhythms. *MTNR1B* rs16918190, located 105 kb upstream of the gene, was identified as having experienced positive selection in East Asia [235]. Although recent reports were unable to find an association between rs16918190 and glucose regulation, the SNP may act as a gene transcriptional control in the regulatory region.

Nevertheless, another meta-analysis showed a consistent genetic effect of *MTNR1B* rs10830963 on glycemic traits in Europeans and African Americans but no positive selection in the locus [121]. Indeed, recent phylogenetic analyses have indicated a conserved evolutionary relationship of *MTNR1B* between humans and vertebrates both in terms of structure and function [1,234,235]; specifically, a synteny analysis found a conserved suite consisting of seven neighboring genes in different species that varied upstream and downstream of *MTNR1B,* which may enhance the conservation of the gene [1]. Thus, it is difficult to determine the evolutionary selection of the “thrifty gene” for T2DM.

T2DM is a disease attributed to polygenic inheritance, environmental factors and behaviors. Although the genetic variants of *MTNR1B* appear to explain only small effect sizes regarding the risk of T2DM, the rs1830963 G allele consistently provides a robust signal for T2DM risk across multiple populations. *MTNR1B* is a critical mediator in melatonin signaling that links circadian rhythms to T2DM, and a common variant of *MTNR1B* rs10830963 seems to further alter the nocturnal release of melatonin, inhibiting its release from β-cells; this alteration leads to a delayed rhythm and elevated glucose levels during the night as well as in the early morning. In terms of the “thrifty gene” hypothesis, it is plausible that harsh survival stressors might have triggered the evolution of adaptive variants of *MTNR1B* rs10830963 that maintained elevated fasting glucose levels to prevent overnight starvation. While this finding of a possible link between the selective pressure on *MTNRIB* and T2DM risk is of great interest, the thrifty mechanisms of *MTNR1B* need further elucidation.

## 6. Conclusion and future perspectives

A great deal of evidence supports the key role of the circadian rhythm in glucose homeostasis. Pineal melatonin is a critical chronobiotic hormone that synchronizes the endogenous circadian rhythm to the exogenous photoperiod. This review highlights the role of melatonin and its receptors in glucose metabolism and T2DM *via* the circadian rhythm. The possible mechanisms include the binding of melatonin to its receptors (MT1 and MT2) in the SCN and peripheral tissues (pancreatic, muscle, liver, adipose and gut), which control glucose metabolism *via* multiple downstream signaling cascades, such as the Kir3 ion channel, PKC, cGMP, and IP3. Furthermore, the findings from GWASs and replicated cohort studies indicate a robust genetic effect of *MTNR1B*, which codes melatonin receptor MT2, on glycemic traits and T2DM risk, especially the rs10830963 G allele. However, there is currently not insufficient functional evidence to determine the effects of *MTNR1B* variants on the risk of T2DM. Evolutionary studies have implied selective pressure on *MTNR1B* and supported its importance in glucose homeostasis. A common variant at *MTNR1B* rs10830963 may lead to later melatonin secretion onset in the dim-light cycle and an extended duration of elevated melatonin levels, further affecting the circadian rhythm and elevating fasting glucose levels as well as increasing insulin resistance. Accordingly, we propose that genetic variants in *MTNR1B* resulted from evolutionary selection to facilitate adaptation to famine or food shortage by maintaining fasting glucose for survival; unfortunately, these variants now confer susceptibility to T2DM. In conclusion, *MTNR1B* may be a “thrifty gene” that is experienced in positive selection to link T2DM *via* circadian misalignment in modern society. The “thrifty gene” hypothesis might indicate a possible mechanism underlying T2DM and provide a novel target for the prevention and treatment of T2DM.

## Data Availability

The authors confirm that the data supporting the findings of this study are available within the review.
